# Human papilloma virus E1-specific T cell immune response is associated with the prognosis of cervical cancer patients with squamous cell carcinoma

**DOI:** 10.1186/s13027-018-0206-5

**Published:** 2018-11-16

**Authors:** Miaomiao Ma, Yaning Feng, Peiwen Fan, Xuan Yao, Yanchun Peng, Tao Dong, Ruozheng Wang

**Affiliations:** 1 Department of Radiation Oncology, The Affiliated Tumor Hospital of Xinjiang Medical University, Ürümqi, China; 2 Key Laboratory of Cancer Immunotherapy and Radiotherapy, Chinese Academy of Medical Sciences, Ürümqi, China; 3 MRC Human Immunology Unit, The Weatherall Institute of Molecular Medicine, Oxford, UK; 4 Nuffeld Department of Medicine, CAMS Oxford Center for Translational Immunology, Chinese Academy of Medical Science Oxford Institute, Oxford University, Oxford, UK

**Keywords:** Human papillomavirus 16, Cervical squamous cell carcinoma, Enzyme-linked immunoassay, T cell immune response, PBMC

## Abstract

**Background:**

Cervical cancer is attributable to human papilloma virus (HPV) infection in the majority cases. E1, an HPV derived-protein, plays an important role in the initiation and development of cervical cancer. Our study aims to investigate the HPV E1-specific T cell response in patients with cervical squamous cell carcinoma (CSCC).

**Methods:**

A total of 66 CSCC patients with FIGO stage IIB-IIIB and 60 healthy controls were enrolled. Enzyme-Linked ImmunoSpot (ELISPOT) assays was used to measure the HPV E1-specific T cell response in the peripheral blood of these patients before treatment. The patients were treated with chemotherapy and/or radiotherapy and followed up clinically for three years. The relationship between the T cell response, various clinical characteristics and the prognosis were studied with univariate analysis, multivariate analysis and survival curve analysis.

**Results:**

The frequency of HPV E1-specific T cell response in peripheral blood of cervical cancer patients was 59.09%, with mean response intensity 24.56 SFC/10^6^ PBMCs. The frequency and intensity of HPV E1-specific T cell response in patients were higher than healthy controls(*p* < 0.001; *p* = 0.009). The intensity of HPV E1-specific T cell responses were higher in the stage IIB patients and patients with no pelvic lymph node metastasis (*p* = 0.038; *p* = 0.044). Univariate analysis showed that HPV E1 specific T cell response was associated with progression-free survival (PFS) and overall survival (OS) (PFS: *p* = 0.021; OS: *p* = 0.004). Multivariate analysis showed that HPV E1-specific T cell response was an independent prognostic factor influencing PFS and OS among all the factors included in our study (PFS: *HR* = 7.252, 95%*CI* = 1.690–31.126, *p* = 0.008; OS: *HR* = 7.499, 95%*CI* = 1.661–33.856, *p* = 0.009). The survival curves showed that the rate of PFS and OS in patients with HPV E1 specific T cell response was significantly higher than those who did not response.

**Conclusions:**

Our study demonstrated that the level of HPV E1-specific T cell response was correlated with the survival of advanced patients with CSCC. Patients who displayed no HPV E1-specific T cell response were more likely to be those with poor prognosis.

## Background

Cervical cancer (CC) is one of the most frequent malignant tumors among women worldwide. The incidence ranked as the second among cancers diagnosed in women [1]. It was estimated that squamous cell carcinoma (SCC) accounts for about 90% of all CCs in Xinjiang, a province located in western China [2–4], while the advanced CCs accounts for more than 60%. Although radiotherapy and chemotherapy are considered to be the most effective strategies, the long-term survival of patients with advanced CC still remains to be improved [5]. Therefore, it is imperative to develop novel therapies to benefit more patients.

Persistent and chronic infection of high-risk types of human papillomavirus (HR-HPV), especially type 16, has been confirmed as the principal risk factor for the initiation and development of SCC [6]. In order to prevent the HPV infection and the mortality caused by HPV-related CCs, vaccines against the HR-HPV have been developed [7]. However, these prophylactic vaccines rarely showed therapeutic effects to established HPV infections or the related cervical cancer. In recent years, researchers have devoted themselves to designing therapeutic HPV vaccines. Several types of therapeutic vaccines, including recombinant protein vaccines, peptide vaccines, chimeric vaccines, nucleic acid vaccines, etc. are being assessed in terms of efficacy and safety [8–10]. However, there is not yet any such therapeutic vaccine licensed [11]. One of the main objective of the therapeutic vaccine is to initiate immune response against HPV proteins expressed on malignant tumour, which can potentially destroy the tumour eventually. T cell is a critical component of anticancer immune response. It is well recognized that T cell recruitment and infiltration into solid tumours are related to survival of various cancers [12]. T cells can suppress tumour growth by releasing a plethora of cytokines and/or inducing tumour cell apoptosis upon stimulation of the specific antigens presented on the surface of tumour cells [13]. Therefore, T cell might hold the promise of immunotherapy against HPV-related CC.

Previous studies have shown that E1 protein is necessary for HPV replication. On the transcription level, E1 can bind to upstream regulatory sequence of HPV to promote the expression of HPV E6 and E7 oncogenes [14, 15]. Some scholars demonstrated that E1 participated in the early stage of carcinogenesis and can activate specific cytotoxic T cell responses [16]. According to previous research considered E6 and E7 proteins are targets for immunotherapy against tumors induced by HPV [17], but the immune response to HPV E1 antigen protein was not reported. Another study also showed the E1 expression can be detected at various stages of CC development where the level was associated with CC prognosis [18].

We employed the enzyme-linked immunospot assay (ELISPOT) to measure the level of HPV E1-specific T cell response in peripheral blood of patients with advanced CC and healthy control subjects. The relationship between T cell response and clinical characteristics as well as the survival of the patients were analyzed so as to provide evidence for the HPV-specific therapeutic vaccine development.

## Methods

### Study subjects

The study was approved by Third Affiliated Hospital of Xinjiang Medical University Ethics Committee. From February 2014 to April 2015, 66 CSCC patients admitted to Affiliated Tumor Hospital of Xinjiang Medical University were enrolled in this study. The cervical cancer was diagnosed with clinical pathology. The age median of the patients was 54 years with the range from 39 to 80 years (Table 1). They were scored no less than 70 with Karnofsky Performance Status [19]. The patients were treatment-naive before admission and had no other types of cancer, autoimmune disease or infectious diseases. They were staged according to 2009 International Union of Gynecology and Gynecology (FIGO) clinical staging criteria, of which 32 cases were stage IIB and 34 cases were stage IIIA-IIIB (Table 1). All patients were HPV16 positive when the exfoliated cervical cells were genotyped with Hybrimax HPV DNA detection method. Meanwhile, sixty healthy women from physical examination center of the same hospital without CC were recruited as controls (aged from 24 to 60 years). None of them were high risk HPV type positive when screened with the same method as the patients. Written informed consent was given from all study subjects.

**Table 1:** The HPV E1-specific T cell response in peripheral blood of healthy controls and patients with cervical squamous cell carcinoma

Factor	HPV E1-specific T cell response	
	Frequency (%)	Mean magnitude (SFC/10^6^)
Healthy controls(*N* = 60)	26.67	9.94
Patients (*N* = 66)	59.09	24.56
*χ*^2^/*Z*	13.433	−2.594
*P*-value	0.000	0.009

### Ex vivo interferon-γ ELISPOT Assay

Ten milliliters venous blood was obtained from each patient on the second day after admission. Peripheral blood mononuleated cells (PBMCs) were isolated from the blood with Lymphoprep (STEMCELL Technologies*, origin*). Interferon-γ (IFN-γ) ELISPOT assay was used to measure HPV E1-specific T cell responses in the freshly isolated PBMCs as mentioned previously [16, 20]. Briefly, PBMCs were stimulated with overlapping peptide pool (Sigma-Aldrich, USA) representing the whole E1 protein in PVDF plates (Millipore, Bedford, MA, USA) pre-coated with 1-DIK monoclonal antibodies (Machete, Stockholm, Sweden) at 2 × 10^5^ PBMCs/well. Meanwhile, PBMCs stimulated with 20 μg/mL phytohemagglutinin (PHA) (Murex Biotech Limited, Dartford, UK) served as positive controls while unstimulated PBMCs were negative controls. After being cultured overnight, spot-forming cells (SFCs) were counted by automated ELISPOT assay reader (AID ELISPOT reader system, Autoimmune Diagnostika GmbH, Strassberg, Germany). Antigen-specific T-cell responses were considered positive when the number of SFCs from the peptide-pulsed well was greater than three times as many as those in negative control [21]. The adjusted SFCs after subtracting the average negative values were expressed as SFC/10^6^ PBMCs.

### Treatment

All patients were treated according to the National Comprehensive Cancer Network (NCCN) guideline of CC. Among them, 38 patients received concurrent radiotherapy and chemotherapy while 28 received radiotherapy but not chemotherapy due to the intolerance. Intracavitary irradiation (high-dose rate brachytherapy with three-dimensional conformal radiation therapy) and/or extracorporeal irradiation (intensity-modulated radiation therapy or three-dimensional conformal radiation therapy) were decided according to the patient’s specific condition. Patients underwent whole-pelvic external beam radiotherapy at a dose of 45–50.4 Gy together with high-dose-rate brachytherapy. The entire para-aortic lymph node was included if the patient was found to have para-aortic lymph node involvement. High-dose-rate brachytherapy was administered at median dose of 30 Gy in 6 fractions with 5 Gy each time. According to their conditions, 57.58% (38/66) patients received DDP (cisplatin at 40 mg/m^2^) per week or TP (taxol 135 mg/m^2^ + cisplatin 50 mg/m^2^) three times during radiotherapy.

### Follow-up

Post-treatment follow-up visits were scheduled at 1 month, 3 months, and then every 3 months for the first 2 years, and then every 6 months for the next 2 years. Either interview through telephone calls or re-examination in hospital were used to determine the patients’ condition during the follow-up. Overall survival (OS) was defined as the time between the date of the end of treatment and the date of CC-related death or the last follow-up. Progression-free survival (PFS) referred to the time from the date when treatment ended to the date of progression of the tumor or the last day of follow-up.

### Statistical analysis

All statistical analyses were performed using SPSS version 17.0 (SPSS Inc., Chicago, IL, USA). The data not fitting normal distribution was analyzed with rank sum test. The count data were analyzed by the Chi-square test. Two-tailed Fisher’s exact test was conducted where Chi-square test was not applicable. OS and PFS were analyzed by the Kaplan-Meier method, the statistical significance of which was determined by log-rank test. The clinical features and the E1-specifc T cell response were included in the multivariate analysis based on the Cox proportional hazard model using the stepwise method. *P* < 0.05 was considered statistically significant.

## Results

### HPV E1-specific T responses in patients with cervical squamous cell carcinoma were stronger than healthy controls

**Fig. 1:**
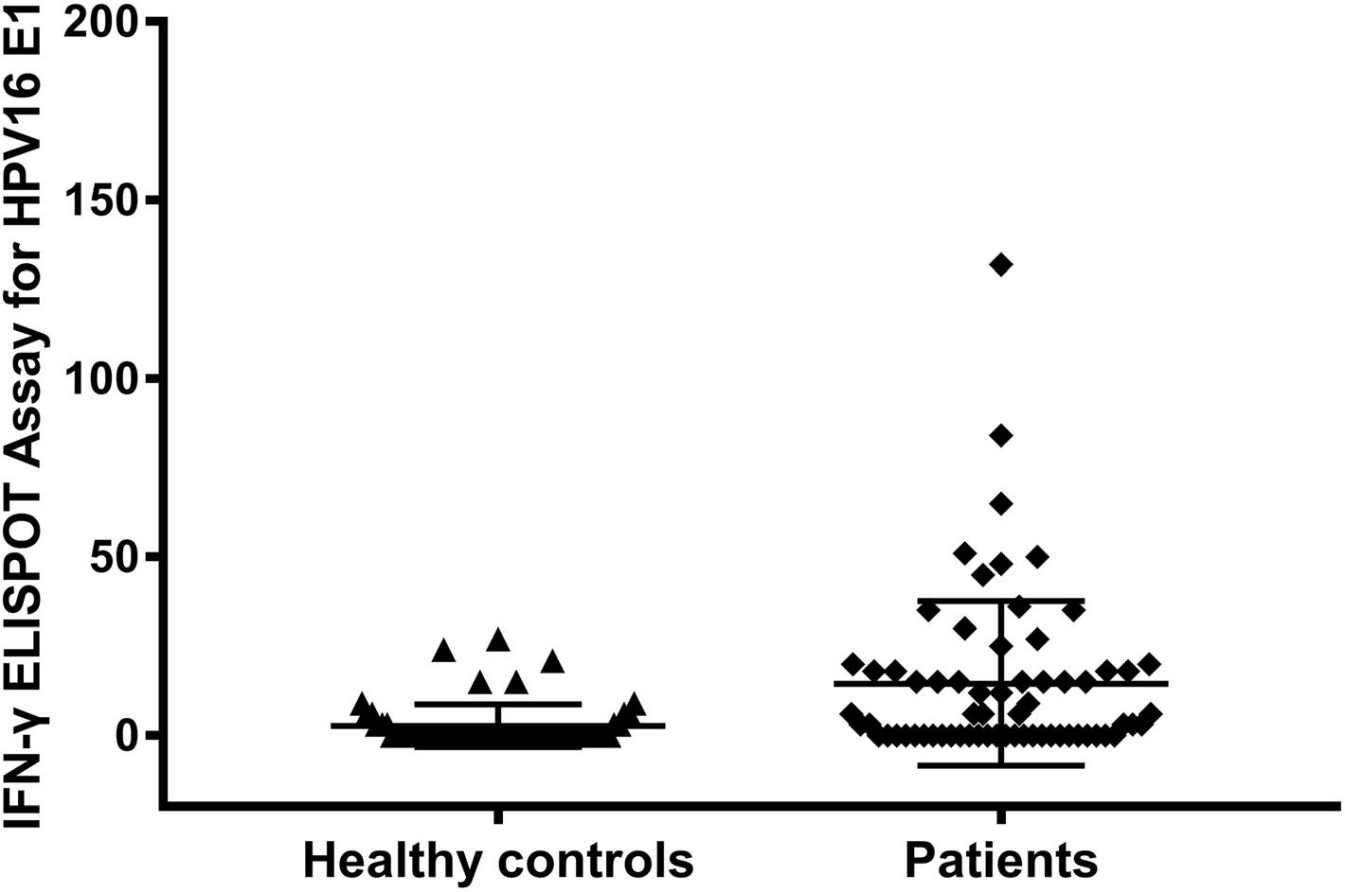
The HPV E1-specific T cell responses in peripheral blood of healthy controls and patients with cervical squamous cell carcinoma by IFN-γELISPOT Assay

The frequency of HPV E1-specific T cell response in peripheral blood of the 60 healthy controls was 26.67% and the mean response intensity was 9.94 SFC/10^6^ PBMCs, while that of the 66 CSCC patients was 59.09% and 24.56 SFC/10^6^ PBMCs, respectively (Fig. 1). The frequency and intensity of HPV E1-specific T response in patients were higher than healthy controls (χ^2^ = 13.433, *p* < 0.001; *Z* = − 2.594, *p* = 0.009). There were statistically significant difference between the two groups (Table 1).

### HPV E1-specific T cell responses differed among patients of stage IIB and IIIB

No significant difference was found in the response frequency when the patients were grouped with age, family history of cancer, abortion history, tumor size, histological type, pathological grade, clinical stage, levels of Squamous cell carcinoma antigen (SCC-Ag), Carcinoembryonic antigen (CEA), Tumor specific growth factor (TSGF), or pelvic lymph node metastasis (Table 2).

**Table 2:** The relationship between response frequency of HPV E1-specific T cell response in peripheral blood and clinical features in patients with cervical squamous cell carcinoma

Factor		*N*	Frequency (%)	*χ* ^2^	*P*-value
Age (year)	≤54	32	53.13	0.915	0.339
	> 54	34	64.71		
Family history of cancer	No	59	57.63	0.678	0.767
	Yes	7	71.43		
History of miscarriage	No	30	56.67	0.134	0.715
	Yes	36	61.11		
Tumor size (cm)	≤5	43	58.14	0.046	0.830
	> 5	23	60.87		
Tumor type	Cauliflower	25	52.00	1.868	0.60
	Nodular	29	58.62		
	Hollow	7	71.43		
	Others	5	80.00		
Histologic grade	Well	2	0	4.465	0.215
	Moderate	45	64.44		
	Poor	13	53.85		
	Papillary	6	50.00		
FIGO stage	IIB	34	59.38	0.002	0.964
	IIIA-IIIB	32	58.82		
SCC-Ag	Normal	12	75.00	1.536	0.215
	Anormal	54	55.56		
CEA	Normal	42	64.29	1.289	0.256
	Anormal	24	50.00		
TSGF	Normal	46	54.35	1.413	0.235
	Anormal	20	70.00		
Pelvic lymph nodes	No	37	64.86	1.161	0.281
	Yes	29	51.72		

*N* number of patients, *FIGO* International Federation of Gynecology and Obstetrics, *SCC-Ag* Squamous cell carcinoma antigen, *CEA* carcinoembryonic antigen, *TSGF* Tumor specific growth factor

It was noted that the intensity of HPV E1-specific T cell responses in stageII group were higher than III group, the differences of which were statistically significant (*Z* = − 2.077, *p* = 0.038). The intensity of HPV E1-specific T cell responses in no pelvic lymph node metastasis group were significantly higher than pelvic lymph node metastasis group (*Z* = − 2.017, *p* = 0.044). However, when comparing T cell response intensity between patients grouped by ethnicities, age, family history of cancer, abortion history, tumor size, histological type, pathological grade, SCC-Ag level or CEA level, TSGF level, no significant difference was found (Table 3).

**Table 3:** The relationship between response intensity of HPV E1-specific T cell response in peripheral blood and the clinical features in patients with cervical squamous cell carcinoma

Factor		*N*	Mean magnitude (SFC/10^6^)	*Z*	*P*-value
Age (year)	≤54	32	20.06	−0.598	0.550
	> 54	34	28.05		
Family history of cancer	No	59	24.74	−0.570	0.568
	Yes	7	23.4		
History of miscarriage	No	30	24.88	−0.570	0.569
	Yes	36	24.32		
Tumor size (cm)	≤5	43	23.4	−0.662	0.508
	> 5	23	26.64		
Tumor type	Cauliflower	25	24.77	−0.115	0.909
	Nodular	29	24.65		
	Hollow	7	24.6		
	Others	5	23.5		
Histologic grade	Well	2	0	−1.826	0.068
	Moderate	45	21.31		
	Poor	13	39.28		
	Papillary	6	21.67		
FIGO stage	IIB	34	27.45	−2.077	0.038
	IIIA-IIIB	32	21.53		
SCC-Ag	Normal	12	23	−0.419	0.675
	Anormal	54	25.03		
CEA	Normal	42	22.56	−1.239	0.215
	Anormal	24	29.08		
TSGF	Normal	46	30.72	−1.958	0.050
	Anormal	20	13.57		
Pelvic lymph nodes	No	37	25.58	−2.017	0.044
	Yes	29	22.93		

*N* number of patients, Mean magnitude of T cell response of the population, *FIGO* International Federation of Gynecology and Obstetrics, *SCC-Ag* Squamous cell carcinoma antigen, *CEA* carcinoembryonic antigen, *TSGF* Tumor specific growth factor

### HPV E1-specific T cell response was related to the prognosis

To see if E1-specifc T cell response was related to CC prognosis, the patients were followed as the protocol mentioned above for a median period of 36 months (range 4 to 48 months). The last follow-up date was May 2018. Two patients were lost and the follow-up rate ended up with 96.97%. Of the 66 patients, 13 died from recurrent and metastatic disease, 1 died from uremia syndrome. The 3-year OS and PFS rates were 79.69 and 76.56%, respectively. Univariate analysis showed that the HPV E1-T cell response was related to the PFS and OS of CC*.* Patients showing E1-specific T cell response displayed significantly improved PFS compared to the non-responders*,* the difference of which was significant (χ^2^ = 5.307, *p* = 0.021; χ^2^ = 8.078, *p* = 0.004). Age was also found to be correlated with the PFS (χ^2^ = 3.963, *p* = 0.047) (Table 4).

**Table 4:** Log-rank analysis of prognostic factors in cervical squamous cell carcinoma patients

Factor		PFS			OS		
		3-year (%)	*χ* ^2^	P-value	3-year (%)	*χ* ^2^	*P*-value
Age (year)	≤54	84.91	3.963	0.047	88.66	2.392	0.122
	> 54	64.52			67.74		
Family history of cancer	No	71.70	1.884	0.170	75.47	1.618	0.203
	Yes	100.00			100.00		
History of miscarriage	No	66.67	2.299	0.129	74.07	0.945	0.331
	Yes	80.33			80.33		
Tumor size (cm)	≤5	73.68	0.027	0.870	78.95	0.085	0.771
	> 5	74.36			74.36		
Tumor type	Cauliflower	76.74	0.214	0.644	76.74	0.033	0.857
	Nodular	72.00			76.00		
	Hollow	57.14			71.43		
	Others	100.00			100.00		
Histologic grade	Well	50.00	0.239	0.625	50.00	0.066	0.797
	Moderate	75.31			80.25		
	Poor	66.67			66.67		
	Papillary	83.33			83.33		
FIGO stage	IIB	82.46	1.271	0.260	82.46	1.166	0.280
	IIIA-IIIB	72.41			65.52		
SCC-Ag	Normal	90.00	1.854	0.173	90.00	1.367	0.242
	Anormal	70.53			74.74		
CEA	Normal	80.56	1.951	0.163	86.00	3.238	0.072
	Anormal	62.79			62.79		
TSGF	Normal	72.84	0.191	0.662	77.78	0.003	0.960
	Anormal	76.47			76.47		
Pelvic lymph nodes	No	79.59	1.698	0.193	79.59	0.571	0.450
	Yes	69.70			75.76		
HPV E1	Positive	85.29	5.307	0.021	91.18	8.078	0.004
	Negative	57.45			57.45		
Treatment	Radiotherapy + chemotherapy	78.13	1.073	0.300	81.25	0.265	0.607
	Radiotherapy	68.63			72.55		

*FIGO* International Federation of Gynecology and Obstetrics, *SCC-Ag* Squamous cell carcinoma antigen, *CEA* carcinoembryonic antigen, *TSGF* Tumor specific growth factor, *HPV* Human papillomavirus

We further analyzed the weight of various factors on the prognosis of patients with cervical cancer. Multivariate Cox regression analysis was performed to determine if survival of the advance CC patients was correlated with any clinical features or E1-specific T cell response. It was found that the presence of E1-specific T cell response was independently correlated with better PFS and OS (Hazard ratio [HR] 7.252, 95% Confidence interval [CI] 1.690–31.126, *p* = 0.008 and *HR* = 7.499, 95%*CI* = 1.661–33.856, *p* = 0.009, respectively). Besides, younger age was observed to be related to better PFS of patients (*HR* = 5.423, 95%*CI* = 1.113–26.419, *p* = 0.036) (Table 5) and no pelvic lymph node metastasis group had better PFS of patients (*HR* = 5.035, 95%*CI* = 1.140–22.231, *p* = 0.033) (Table 5).

**Table 5:** Cox regression analyses of progression-free survival in cervical squamous cell carcinoma patients

Factor	PFS		OS	
	HR (95%CI)	*P*-value	HR (95%CI)	*P*-value
Age	5.423 (1.113–26.419)	0.036	4.265 (0.901–20.194)	0.067
Family history of cancer	–	–	–	–
History of miscarriage	0.521 (0.150–1.809)	0.304	0.735 (0.208–2.600)	0.634
Tumor size	1.629 (0.443–5.598)	0.463	2.310 (0.571–9.345)	0.240
Tumor type	0.947 (0.400–2.239)	0.901	0.594 (0.194–1.820)	0.362
Histologic grade	0.922 (0.374–2.271)	0.860	0.772 (0.291–2.045)	0.602
FIGO stage	1.031 (0.221–4.804)	0.969	0.934 (0.196–4.450)	0.931
SCC-Ag	0.720 (0.067–7.774)	0.787	0.931 (0.086–10.035)	0.953
CEA	0.766 (0.194–3.015)	0.702	1.902 (0.342–10.581)	0.463
TSGF	0.381 (0.096–1.515)	0.171	0.657 (0.154–2.808)	0.571
Pelvic lymph nodes	5.035 (1.140–22.231)	0.033	2.844 (0.571–14.166)	0.202
HPV E1-specific T cell response	7.252 (1.690–31.126)	0.008	7.499 (1.661–33.856)	0.009
Treatment	1.595 (0.532–4.784)	0.405	0.118 (0.347–3.603)	0.851

*FIGO* International Federation of Gynecology and Obstetrics, *SCC-Ag* Squamous cell carcinoma antigen, *CEA* carcinoembryonic antigen, *TSGF* Tumor specific growth factor, *HPV* Human papillomavirus

### Kaplan–Meier survival curve analysis revealed the association of HPV E1-specific T cell response with survival

**Fig. 2:**
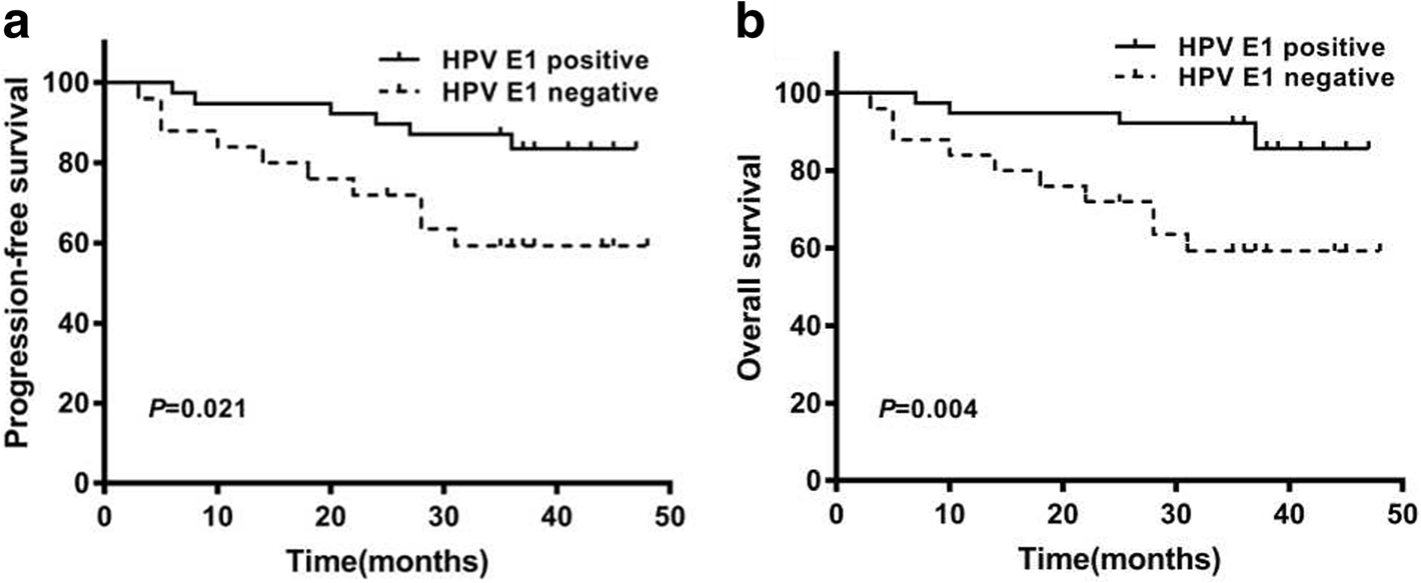
Survival difference between cervical squamous cell carcinoma in patients with or without HPV E1-specific T cells response. **a** Kaplan-Meier plot for progression-free survival (PFS) analysis of advanced CC patients with or without HPV E1-specific T cell response. **b** Kaplan-Meier plot for overall survival (OS) analysis of advanced CC patients with or without HPV E1-specific T cell response

Kaplan–Meier analysis was used to estimate the survival difference between E1-specific responders and non-responders. The survival curves demonstrated that presence of E1-specific T cell response was associated with significantly higher PFS and OS (Log-rank value (Mantel–Cox) = 5.307, *p* = 0.021; Log-rank value (Mantel–Cox) = 8.153, *p* = 0.004, respectively) (Fig. 2). In other words, patients displaying HPV E1-specific T cell response had significantly improved PFS and OS.

## Discussion

In recent years, with accumulating knowledge of the relationship between HPV and CC [22, 23], researchers have invested a great amount of effort to develop immunotherapies that can effectively treat CC. It is believed that T cells, an important part of adaptive immune response, play major roles in suppressing and eliminating HPV-infected cells [24–26]. Therefore, exploiting the curative potential of T cells can benefit CC patients who are not well treated with conventional methods such as chemotherapy and radiotherapy.

Our previous study reported that the frequency of HPV E1-specific T response was higher in patients with head and neck squamous cell carcinoma than in healthy controls [27]. Since HPV contributes to the development of CC, we explored whether E1-specific immune response existed in CC patients. In this study, we found that CC patients displayed stronger E1-specific T cell response than healthy controls. The HPV16 infection of study subjects among different groups were an important reason for the inconsistency.

HPV E1-specific T cell responses were observed in some healthy controls. One explanation was that these subjects were previously infected by HPV16 but later the virus was cleared by the immune system in a relatively short time [28]. The T cell response was elicited by the memory T cells due to the infection. It is also noteworthy that not all patients had E1-specific T cell response. An important factor that may affect the T cell response is HPV DNA physical status. It was reported that in advanced CC, HPV DNA tends to integrate into host DNA, often resulting in disruption of HPV16 E1 open reading frames thus absence of E1 protein expression [29]. Therefore, E1-specific T cell response cannot be observed in every patient.

We also studied the relationship between HPV E1-specific T cell response in CC patients and their prognosis during a four-year follow up. The correlation between positive E1-specific T cell response and better PFS and OS was found when using univariate analysis (Table 3). Mulitvariate analysis revealed that both the E1-specific T cell response and age influenced the PFS and OS following chemoradiotherapy in advanced CC patients (Table 4). It was also worth to mention that generally those whose T cell responded to E1 stimulation had better survival, suggesting the anticancer function of the T cell in cervical cancer.

The conventional treatment modality like radiotherapy and chemotherapy are currently the most commonly used to treat advanced CCs. However, the long-term survival of advanced CC is still low [5, 30]. To improve the survival, novel immunotherapies are being developed. T cells are versatile in suppressing cancer development. Upon stimulation with the antigen, T cell can either disrupt cancer cell function by releasing cytokines or directly kill the cancer cells by inducing apoptosis. Therefore, it is worthwhile to develop T cell-based immunotherapies to fight against advanced CCs. Vaccines have been developed against various HPV16-derived epitopes [31]. Rahma et al. [32] reported the immunogenicity of two epitopes from HPV16 E6 and E7 in advanced CC patients, with response rates 63% (10/16) and 58% (7/12), respectively. The study proved the feasibility of therapeutic vaccines against HPV derived-epitopes. But the problem remained that E6 or E7 cannot initiated immune response in every patient from the above report. Thus, in our study, we looked into the possibility to target HPV E1 to broaden the application to CC patients. According to our result, T cell response against E1 protein can be observed in a subgroup of advanced CC patients. Moreover, the stronger T cell response was related to better prognosis. It can be seen that those patients can benefit from the E1-specific T cell response, which served as an evidence to develop immunotherapy.

However, there are several limitations to this study. Firstly, the T cell response to E1 was only measured in advanced CC patients whereas it remained to be determined how T cell respond in patients staged other than II or III. Secondly, since PBMCs instead of isolated T cell subsets were used in ELISPOT assay, it was not possible to tell which T cell subsets were responsible for the E1-specific response since both CD4+ and CD8+ T cell can secret IFN-γ. Besides, it would be informative if HPV16 negative patients were included in our study. However, HPV16-negative patients were rare and often they were diagnosed with cervical adenocarcinoma, which display different clinical features to CSCC.

## Conclusions

In conclusion, the characteristics of HPV E1-specific T cell immune response in peripheral blood of patients with CC was significantly related to the clinical characteristics and prognosis. It is worthwhile to investigate the potentiality of E1 as an immunotherapy target in the future so as to provide evidence for the HPV-specific therapeutic vaccine development.

## Abbreviations

CEA: Carcinoembryonic antigen
CI: Confidence interval
CSCC: Cervical squamous cell carcinoma
FIGO: Federation of Gynecology
HR: Hazard ratio
HR-HPV: High risk human papillomavirus
OS: Overall survival
PFS: Progression-free survival
SCC-Ag: Squamous cell carcinoma associated antigen
TSGF: Tumor specific growth factor
